# The Radiological Feature of Anterior Occiput-to-Axis Screw Fixation as it Guides the Screw Trajectory on 3D Printed Models: A Feasibility Study on 3D Images and 3D Printed Models

**DOI:** 10.1097/MD.0000000000000242

**Published:** 2014-12-02

**Authors:** Ai-Min Wu, Sheng Wang, Wan-Qing Weng, Zhen-Xuan Shao, Xin-Dong Yang, Jian-Shun Wang, Hua-Zi Xu, Yong-Long Chi

**Affiliations:** From the Department of Orthopaedics, Second Affiliated Hospital of Wenzhou Medical University, Zhejiang Spinal Research Center, Wenzhou, Zhejiang, People's Republic of China (A-MW, SW, W-QW, Z-XS, J-SW, H-ZX, Y-LC); and Department of Anatomy, Wenzhou Medical University, Wenzhou, Zhejiang, People's Republic of China (X-DY).

## Abstract

Anterior occiput-to-axis screw fixation is more suitable than a posterior approach for some patients with a history of posterior surgery. The complex osseous anatomy between the occiput and the axis causes a high risk of injury to neurological and vascular structures, and it is important to have an accurate screw trajectory to guide anterior occiput-to-axis screw fixation.

Thirty computed tomography (CT) scans of upper cervical spines were obtained for three-dimensional (3D) reconstruction. Cylinders (1.75 mm radius) were drawn to simulate the trajectory of an anterior occiput-to-axis screw. The imitation screw was adjusted to 4 different angles and measured, as were the values of the maximized anteroposterior width and the left-right width of the occiput (C0) to the C1 and C1 to C2 joints. Then, the 3D models were printed, and an angle guide device was used to introduce the screws into the 3D models referring to the angles calculated from the 3D images.

We found the screw angle ranged from α1 (left: 4.99 ± 4.59°; right: 4.28 ± 5.45°) to α2 (left: 20.22 ± 3.61°; right: 19.63 ± 4.94°); on the lateral view, the screw angle ranged from β1 (left: 13.13 ± 4.93°; right: 11.82 ± 5.64°) to β2 (left: 34.86 ± 6.00°; right: 35.01 ± 5.77°). No statistically significant difference was found between the data of the left and right sides. On the 3D printed models, all of the anterior occiput-to-axis screws were successfully introduced, and none of them penetrated outside of the cortex; the mean α4 was 12.00 ± 4.11 (left) and 12.25 ± 4.05 (right), and the mean β4 was 23.44 ± 4.21 (left) and 22.75 ± 4.41 (right). No significant difference was found between α4 and β4 on the 3D printed models and α3 and β3 calculated from the 3D digital images of the left and right sides.

Aided with the angle guide device, we could achieve an optimal screw trajectory for anterior occiput-to-axis screw fixation on 3D printed C0 to C2 models.

## INTRODUCTION

Various posterior occipitocervical fusion techniques have been reported,^1,2^ which are based on posterior wires, rods, plates, and screws,^3–6^ and these technique achieve good osseous fusion and have been widely used by spine surgeons.

In a number of clinical situations, such as in the case described by Dvorak et al^7^ patients with a history of posterior surgery leading to disruption of osseous anatomy and significant posterior scar tissue, there are not sufficient landmarks to achieve safe and effective posterior stabilization; Dvorak et al^7^ performed anterior occiput-to-axis screw fixation on the patient in their case study. Simultaneously, a biomechanical study showed that anterior occiput-to-axis screw fixation could achieve biomechanical stability comparable to that of posterior approaches.^8^

Compared to posterior surgery, which causes considerable damage to the extensor muscles as well as bleeding, anterior occiput-to-axis screw fixation could be performed by a percutaneous approach,^9^ which has the advantages of being minimally invasive, causing less blood loss, and resulting in shorter hospital stays.

The following additional advantages of occiput-to-axis screw fixation are reported: the anterior approach could be performed more conveniently than the posterior approach; the supine position is better for reduction and stable recovery and prevents having to change the position after anesthesia; and the anterior approach might be more suitable for patients with pressure from anterospinal compression.

The complex osseous anatomy between the occiput and the axis causes a high risk of injury to the neurological and vascular structure; to avoid these complications, it is important to have an accurate screw trajectory to guide the anterior occiput-to-axis screw fixation.

## METHODS AND MATERIALS

The research was performed following the Declaration of Helsinki principles and was approved by the Institutional Review Board of The Second Affiliated Hospital of Wenzhou Medical University. Informed consent was obtained from the participants.

Thirty computed tomography (CT) scans of upper cervical spines were obtained from the Star PACS system (INFINITT, Seoul, South Korea) of our hospital; the participants were without spinal diseases, and had CT scans for health testing or had presented with oral diseases; patients with any spinal abnormality such as a fracture or tumor were excluded.

The data from the CT scans (in DICOM format) were imported into Mimics software v10.01 (Materialise, Leuven, Belgium) for the three-dimensional (3D) reconstruction. We drew a cylinder (1.75 mm radius) to simulate the trajectory of the anterior occiput-to-axis screw.

**FIGURE 1:**
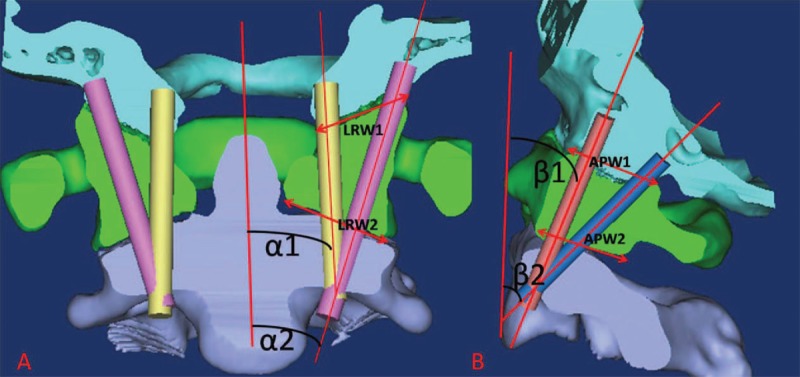
The schematic diagram (A and B) showing the methods of measurements for: α1: maximized interior angle on the AP view; α2: maximized lateral angle on the AP view; β1: minimized dorsal angle on the lateral view; β2: maximized dorsal angle on the lateral view; APW1 and LRW1: anteroposterior width and left-right width of the occiput (C0) to the C1 joint; APW2 and LRW2: anteroposterior width and left-right width of the occiput to the C1 to C2 joint.

The screw entry point was identified at the concavity on the anterior cortex of the C2 arch referred to by Lu et al^10^ and the angle of the imitation screw was adjusted to 4 different angles with the following measurements: maximized interior angle (α1) and maximized lateral angle (α2) on the anteroposterior (AP) view, minimized dorsal angle (β1), and maximized dorsal angle (β2) to the referential line on the lateral view (Figure 1). The values of the maximized anteroposterior width and the left-right width of the occiput (C0) to the C1 (APW1 and LRW1) and C1 to C2 joints (APW2 and LRW2) were measured as well (Figure 1).

The average of α1 and α2 was calculated and is referred to α3, and the average of β1 and β2 was calculated and named β3. Simultaneously, the 3D images data were saved in. STL format and imported to Cura software. After a 3D digital model was created in Cura, we save it in G code format and imported it to a 3D Printer (3D ORTHO Waston Med, Inc., Changzhou, Jiangsu, China) to print the 3D model.

**FIGURE 2:**
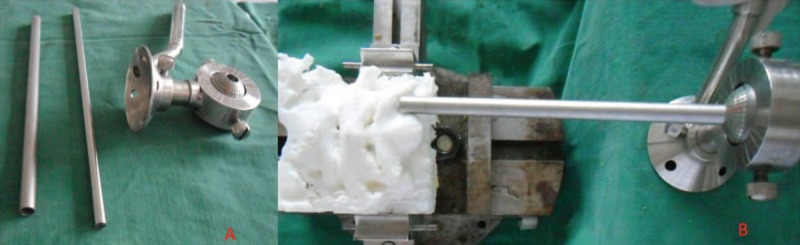
The photo of the angle guide device (A) and the method used to introduce the anterior occiput-to-axis screws on 3D printed models (B).

After the 3D model was printed, it was fixed in a holding device, and an angle guide device was used (Figure 2, Designed by YLC and SW); the K-wire angle was referred to α3 and β3, as previously calculated. After the K-wire was inserted with the assistance of the angle guide device, the AP and lateral X-ray films were obtained, the angle of the screw on the AP and lateral films were measured and referred to as α4 and β4, respectively. Then, the α4 and β4 measured in the 3D models were compared to α3 and β3.

## STATISTICAL ANALYSIS

The statistical analysis was conducted using SPSS software v17.0 (SPSS, Inc., Chicago, IL). A paired *t*-test was used to compare the measurements of the left and right side and the measurements between the 3D digital images and the 3D printed models. The level of significance was set at *P* < 0.05.

## RESULTS

On the AP view, the screw angle ranged from α1 (left: 4.99 ± 4.59°; right: 4.28 ± 5.45°) to α2 (left: 20.22 ± 3.61°; right: 19.63 ± 4.94°); on the lateral view, the screw angle ranged from β1 (left: 13.13 ± 4.93°; right: 11.82 ± 5.64°) to β2 (left: 34.86 ± 6.00°; right: 35.01 ± 5.77°). The anteroposterior width of the C0 to C1 facet was 16.87 ± 1.34 (left) and 16.88 ± 1.38 (right), slightly larger than that at the C1 to C2 facet (left: 15.76 ± 1.53) and 15.94 ± 1.56 (right); the left-right width of the C0 to C1 facet was 12.43 ± 1.93 (left) and 12.04 ± 2.17 (right), shorter than that at the C1 to C2 facet (left: 15.92 ± 2.26) and 16.18 ± 2.00 (right); they were all wide enough for 3.5 mm or 4.0 mm diameter anterior occiput-to-axis screw fixation. No statistically significant difference was found between the data of the left side and right side (Table 1).

**TABLE 1:**
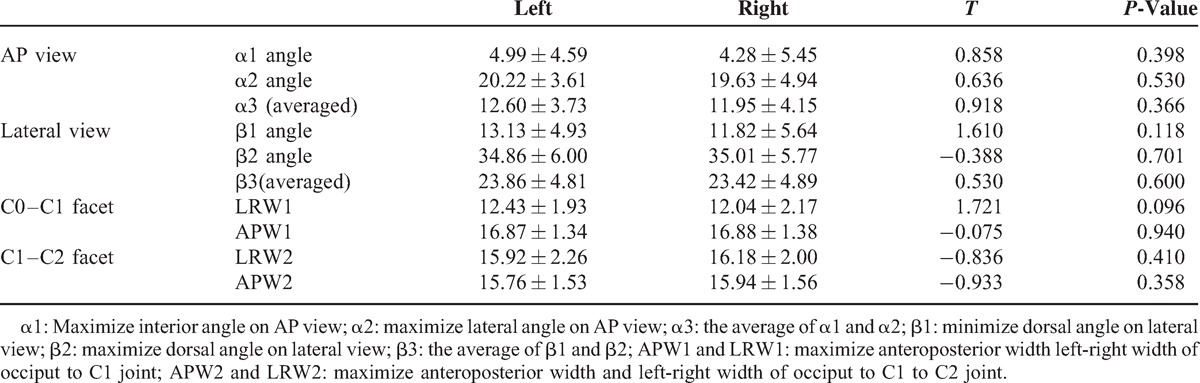
The Parameters Measured From 3D Digital Images

**FIGURE 3:**
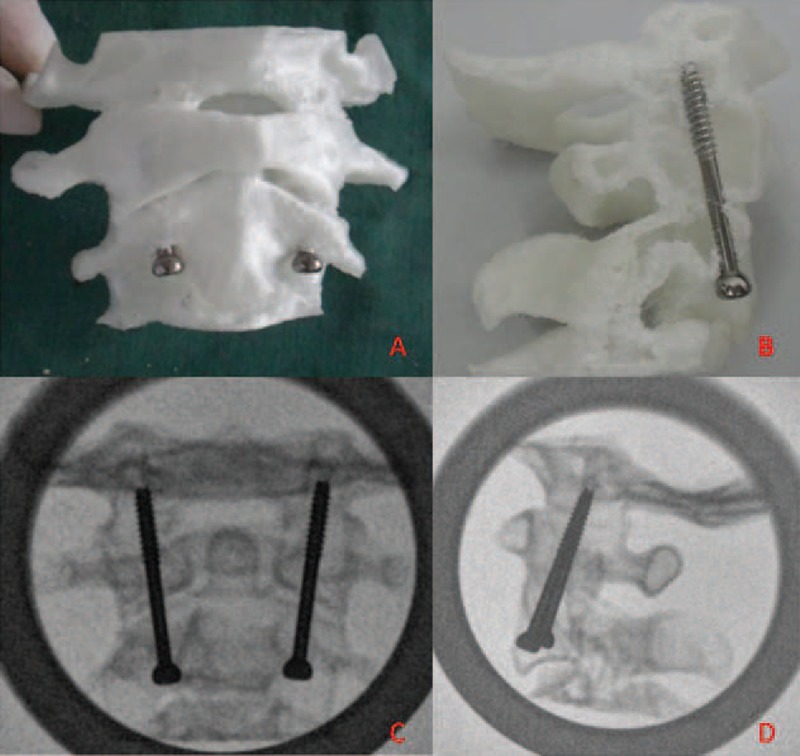
The photo of the 3D printed models after the anterior occiput-to-axis screws were introduced (A and B), the AP (C) and lateral (D) X-ray films of the 3D printed models after the anterior occiput-to-axis screws were introduced.

Guided by the angles of α3 and β3 calculated from the 3D digital images and assisted with the angle guide device, all of the anterior occiput-to-axis screws were successful introduced and none of them penetrated outside of the cortex (Figure 3); the mean α4 was 12.00 ± 4.11 (left) and 12.25 ± 4.05 (right) and the mean β4 was 23.44 ± 4.21 (left) and 22.75 ± 4.41 (right). No significant difference was found between the α4 and β4 from the 3D printed models and the α3 and β3 calculated from the 3D digital images on the left and right sides (Table 2).

**TABLE 2:**
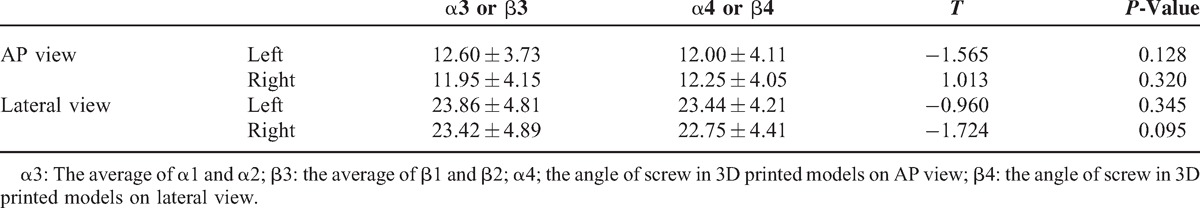
The Comparison of Angles Between 3D Digital Images and 3D Printed Models

## DISCUSSION

Occipitocervical fixation has been challenging for spinal surgeons because of the complex anatomical structures and the high risk of neural and vascular injury at the occipitocervical junction.^11,12^ Most previous reports focused on posterior techniques based on rods, plates, and screws to achieve occipitocervical fusion.^3,4,6^

Posterior stabilization is not suitable in a number of unique situations, such as that of patients with a history of posterior surgery leading to disruption of the osseous anatomy and significant posterior scar tissue, described by Dvorak et al^7^ and that of patients with a history of posterior cranial fossa decompression surgery or dysplastic C2 pedicles, described by Wu et al^9^ Anterior occiput-to-axis screw fixation could be performed via the Smith–Robinson approach or percutaneously; compared to the posterior approach, which causes considerable damage to the extensor muscles and bleeding, the anterior approach is less invasive and results in less blood loss. Cai et al^13^ reported a novel anterior occiput-to-axis locking plate system, which might have a better biomechanical property than simply 2 anterior occiput-to-axis screws; however, their technique is based on 2 anterior occiput-to-axis screws. It is important to analyze the anatomic parameters of anterior occiput-to-axis screw fixation and to establish an accurate method of guiding the optimal screw trajectory.

With the data from the CT scans, we could reconstruct 3D digital images and use a cylinder to imitate the screw trajectory.^14^ Puchwein et al^15^ performed morphometry of the odontoid peg on 3D digital images, and their data were valuable for guiding anterior odontoid screw fixation and informing surgeons on the selection of 1 or 2 anterior odontoid screws.

Our previous study showed that 3D printed models accurately shaped the 3D digital images (data not shown in this study) and is suitable for spinal fixation research. In our study, we used the α3 and β3 calculated from the 3D digital images as the reference and the angle guide device to introduce the anterior occiput-to-axis screws. We found that our method could guide an optimal screw trajectory, and none of the screws in this study penetrated outside of bone cortex.

CT-based navigation was developed in recent decades,^16,17^ and its use could enable accurate screw placement; however, the navigation system is excessively expensive for most hospitals. A worldwide survey found that^18^: despite widespread distribution of the navigation system in North America and Europe, 11% of surgeons use these systems routinely; the lack of equipment, inadequate training, and high costs were the major reasons that the nonusers do not use the navigation systems. The rate of having and using navigation systems in developing countries was less than that in developed countries. Our novel angle guide device is much less expensive than the navigation system, with simple construction and methodology that is relatively easy to learn. The device has potential value for surgeons who do not have access to the expensive navigation system. Further study of this novel angle guide device in clinical practice is needed.

## CONCLUSION

There was enough space for anterior occiput-to-axis screw fixation, and by referring to the parameters of optimal screw trajectory and with the aid of the angle guide device, we could achieve an optimal screw trajectory on a 3D printed C0 to C2 model.
